# Can you FORCE research implementation? A PERUKI survey to evaluate knowledge translation of the FORCE study

**DOI:** 10.1302/2633-1462.79.BJO-2025-0298.R1

**Published:** 2026-09-08

**Authors:** Marianne Jenkins, Jack F. Bennett, Damian Roland, Daniel C. Perry

**Affiliations:** 1 Clinical Lead for Paediatric Emergency Department, Emergency & Acute Medicine Directorate, Cardiff & Vale University Health Board, Cardiff, UK; 2 NHS University Hospitals of Liverpool Group, Liverpool, UK; 3 University of Liverpool, School of Medicine, Liverpool, UK; 4 System Clinical Director, Urgent and Emergency Care, Leicester, Northampton & Rutland Integrated Care System, Leicester, UK; 5 University Hospitals of Leicester NHS Trust, Leicester Royal Infirmary, Leicester, UK; 6 Liverpool Institute of Child Health and Wellbeing, University of Liverpool, Institute in the Park, Alder Hey Hospital, Liverpool, UK; 7 Kadoorie Institute for Trauma and Emergency Care, University of Oxford, Oxford, UK; 8 Department of Orthopaedics, Alder Hey Children's Hospital, Liverpool, UK

**Keywords:** Torus fracture, knowledge translation, dissemination, implementation science, clinicians, torus fractures, FOrearm Fracture, immobilization, plaster casts, Chi-squared test, Fisher’s exact test, clinical trials, splint, torus fractures of the distal radius

## Abstract

**Aims:**

To assess the uptake of the Forearm Fracture Recovery in Children Evaluation (FORCE) trial findings into clinical practice, and to identify the influence of targeted dissemination tools on implementation.

**Methods:**

A cross-sectional electronic survey was distributed via the Paediatric Emergency Research in the UK and Ireland (PERUKI) network. The survey captured data on clinician awareness of the FORCE trial, current management of paediatric torus fracture, the use of dissemination materials, and perceived barriers and enablers to implementation.

**Results:**

A total of 113 responses were analyzed. Most respondents (84.5%) were aware of the FORCE trial. Following publication of the findings, 54.0% reported managing torus fractures in line with trial recommendations – offering a soft bandage or no immobilization, compared with 1.8% prior to the trial. Clinicians involved in delivering the trial were significantly more likely to adopt the recommended management (p = 0.021) and follow-up approach (p = 0.004). Of respondents, 43.4% reported encountering barriers to implementation, the most common being colleague or parent expectations. Peer-to-peer influence (40.8%) and the use of FORCE dissemination tools (38.8%) were the most frequently cited enablers. Among those using the dissemination tools, 79.5% implemented the recommended management compared with 18.2% who did not (p < 0.001).

**Conclusion:**

The findings of the FORCE trial have been adopted across paediatric Emergency Departments in the UK, representing a substantial shift from pretrial practice. Active involvement in research, peer influence, and practical dissemination tools were key drivers of this change. Publication alone appeared insufficient; implementation required engagement, ownership, and resources designed for real-world use. When these were present, practice changed most consistently - supporting the theory that you can FORCE research implementation.

Cite this article: *Bone Jt Open* 2026;7(9):1172–1177.

## Introduction

Evidence-based medicine is the cornerstone of delivering high-quality patient care. However, the process of moving new research findings into clinical practice remains a persistent challenge.^1^ The gap between discovery and implementation has been widely recognized by funders. For example, the National Institute for Health and Care Research (NIHR) now requires all funding proposals to include explicit strategies for implementation and dissemination.^2^ This shift reflects a growing consensus that generating new knowledge is only part of the task; ensuring it is adopted in clinical practice is equally critical.

The FOrearm Fracture Recovery in Children Evaluation (FORCE) study was a multicentre equivalence trial investigating the management of torus (buckle) fractures in children.^3^ The trial was initiated in response to a National Institute for Health and Care Excellence (NICE) research recommendation, following inconclusive evidence from a Cochrane Review of forearm fracture management.^4,5^ Conducted across 23 hospitals and completed in 2020, FORCE found that managing these injuries with a soft removable bandage and immediate discharge from the Emergency Department (ED) was equivalent in terms of pain outcomes to rigid immobilization. This simplified approach was also found to be cost-effective and safe.

The dissemination of findings began in 2022 through a number of channels: conference presentations; academic publications; podcasts; social media; and a dedicated multimedia dissemination toolkit.^6^ The toolkit included an animated video, a downloadable template pathway, an infographic, and a modifiable patient/parent leaflet translated into eight languages. Information regarding the study and dissemination materials can be found in the Supplementary Material. These materials aimed not only to increase awareness and understanding of the trial findings but also to support their integration into clinical practice.

Although traditional dissemination routes, such as journal articles and conferences, remain important, more targeted, accessible, and interactive resources are increasingly recognized as best practice.^7,8^ Their role in supporting implementation, particularly among clinicians, is not well established.

While the efficacy of interventions is frequently studied, what prompts clinicians to change entrenched practice remains less well understood. This study sought to both measure the uptake of the FORCE findings and explore which factors meaningfully influenced clinicians to change their clinical practice concerning this injury.

## Methods

This was a cross-sectional survey conducted via the Paediatric Emergency Research in the UK and Ireland (PERUKI) network, a collaborative group of clinicians and researchers from 76 Paediatric EDs (PEDs) across the UK and Ireland.^9^ Many PERUKI sites had previously participated in the FORCE trial.

### Survey development and distribution

An electronic survey was developed and hosted using REDCap (Research Electronic Data Capture) at Queen’s University of Belfast.^10^ The survey included a combination of multiple-choice and free-text questions designed to explore: awareness of the FORCE study; current management and follow-up of torus fractures; barriers to and enablers of implementation; use and perceived value of the FORCE dissemination materials; and prior involvement in the FORCE trial.

Participants were also invited to provide free-text comments on their experience of implementing the study findings in practice. This survey can be found in the Supplementary Material.

The survey was distributed by email to members of the PERUKI network, including site leads and steering committee representatives. It was open during the winter and spring. To promote engagement, the survey was also featured in the PERUKI seasonal newsletter. The mailing list included 880 individual contacts.

### Statistical analysis

Incomplete submissions were excluded. Descriptive statistics were generated using Excel v. 16.9 (Microsoft, USA). Inferential analysis was performed using SPSS Statistics v. 29.0.20 (IBM, USA). Pearson’s chi-squared or Fisher’s exact test was used to examine associations between categorical variables. A p-value < 0.05 was considered significant.

Free-text responses from three questions were thematically analyzed by two researchers (JFB, MJ) using an inductive coding approach to identify key themes.

### Ethical considerations

Participation in the survey was voluntary. Participants were offered a certificate of completion for their records. The survey was reviewed and approved by the PERUKI executive committee. NHS Research Ethics Committee approval was not required in accordance with UK Health Research Authority guidelines.^11^ Data collection and handling adhered to the General Data Protection Regulation (GDPR).^12^

## Results

A total of 118 responses were initially received. After screening, two responses were removed as they were incomplete. The overall response rate was low at 13.2% with 118 responses from an email list of 880; however, it represented data from 47 of the 76 PERUKI sites (61.8%) across the UK and Ireland. Of these PERUKI sites, the FORCE study recruited through 23 of them (48.9%).

### Awareness of the FORCE study

Most respondents indicated awareness of the FORCE study (n = 98, 84.5%). However, a small proportion stated they were only somewhat aware (n = 15, 12.9%) or had no awareness (n = 3, 2.6%). The full survey was unable to be answered by the three participants with no awareness, resulting in 113 final participants completing the full survey.

Participants were exposed to the study findings from several sources, including reading the publication (76, 67.3%), word of mouth from colleagues (n = 61, 54.0%), a conference presentation (n = 53, 46.9%), social media/online (n = 33, 29.2%), and working or recruiting to the study (n = 29, 25.7%). More than half had exposure from two or more sources (n = 70, 61.9%).

### Implementation of the FORCE study

Prior to the FORCE study publication, most respondents used splint immobilization to manage patients (n = 95, 84.1%), with a small number offering a plaster cast (n = 16, 14.2%), a bandage (n = 1, 0.9%), or no treatment (one, 0.9%). After the publication and dissemination of the FORCE study, the most common treatment was to still offer splint immobilization (n = 52, 46.0%), followed by offering a bandage (n = 42, 37.2%) or no treatment (n = 19, 16.8%), with no participants reporting plaster cast immobilization. In total, over half of the participants were managing patients in line with the FORCE study’s conclusion of offering a bandage or no treatment (n = 61, 54.0%). Clinicians who had worked on or recruited to the study were significantly more likely to adopt these findings (Table I).

**Table I.** Torus fracture management and follow-up adherence to FORCE recommendations by whether the respondent worked on or recruited to the study.

	Worked on or recruited to study, n (%)		
Variable	Yes*	No*	p-value†
**Torus fracture management**			
Bandage or no treatment (FORCE recommendation)	21 (72.4)	40 (47.6)	0.021
Other	8 (27.6)	44 (52.4)	
**Torus fracture follow-up**			
No follow-up (FORCE recommendation)	28 (96.6)	59 (70.2)	0.004
Other	1 (3.4)	25 (29.8)	

* Percentages reported out of the number of the participants in each sub-group (‘Yes’ or ‘No’) of those who worked on or recruited to the study.

† Chi-squared test.

FORCE, FOrearm Fracture Recovery in Children Evaluation.

When asked about follow-up arrangements for torus fractures, most reported that they offer no follow-up in line with FORCE recommendations (n = 87, 77.0%), with the remainder reporting they offered a virtual fracture clinic appointment (n = 23, 20.4%) or an in-person fracture clinic led by Trauma & Orthopaedics (n = 2, 1.8%) or the ED (n = 1, 0.9%). Those who had worked on or recruited to the study were significantly more likely to follow the FORCE recommendation of offering no follow-up (Table I).

Participants were asked whether their management of torus fractures had changed because of the study. In total, 46 respondents (40.7%) indicated it had “completely changed” their practice, while 48 (42.5%) indicated it had “somewhat changed” their practice and 19 (16.8%) indicated that they were *“*still doing what I have always done”.

### Barriers to the implementation of the FORCE study

Just under half of respondents reported they faced barriers to changing practice (n = 49, 43.4%). Of those who reported barriers, patients/carers were the most encountered barrier, followed by medical colleagues within ED and outside of ED. Peer-to-peer influence and the FORCE dissemination materials were the most frequently used methods to overcome these barriers (Table II).

**Table II.** Barriers to changing practice and methods to overcoming them.

Barriers	N (%)*
**What were the barriers/challenges to changing practice?**	
Parents/carers perceptions	17 (34.7)
Clinical colleagues (within ED)	16 (32.7)
Clinical colleagues (outside of ED)	16 (32.7)
Department/organization	13 (26.5)
Nursing colleagues	12 (24.6)
Other	8 (16.3)
**How did you overcome the barriers?**	
Peer-to-peer influence	20 (40.8)
Using FORCE dissemination tools for education	19 (38.8)
Other	18 (36.7)
Doctor’s education	17 (34.7)
Nursing education	14 (28.6)
Multiprofessional departmental teaching	11 (22.4)

* Percentages reported out of the total number of respondents who reported they faced barriers (n = 49).

ED, Emergency Department; FORCE, FOrearm Fracture Recovery in Children Evaluation.

The most reported free-text theme was the time to write, update, and gain approval for organizational guidelines. Some respondents highlighted the influence of “guideline ownership”, with several citing greater delays and resistance to change when the guideline was led by the orthopaedic team, rather than the ED team. It was also frequently highlighted that non-medical individuals, especially support workers who applied plaster casts, were part of the resistance to change.

In response to the barriers, it was reported that “discussion with parents” and “education to parents and families” helped overcome them. In particular, “exploration of concerns and providing explanations and rationale” was noted to be successful in reassuring families that the FORCE findings were equivalent and safe for their child.

### Use of dissemination resources

The majority of participants were aware of the FORCE dissemination materials (n = 61, 54.0%). Among those with awareness, many stated that they or their department used the dissemination materials (n = 39, 63.9%). Furthermore, of those who used the dissemination resources, a high proportion reported sharing them with patients (n = 32, 82.1%).

Those who reported use of the dissemination materials were highly likely to manage torus fractures in line with the FORCE study recommendation (Table III).

**Table III.** Torus fracture management and follow-up adherence to FORCE recommendations by whether the respondent used FORCE dissemination materials of those who were aware of them.

	Use FORCE dissemination materials, n (%)		
Variable	Yes*	No*	p-value
**Torus fracture management**			
Bandage or no treatment (FORCE recommendation)	31 (79.5)	4 (18.2)	< 0.001†
Other	8 (20.5)	18 (81.8)	
**Torus fracture follow-up**			
No follow-up (FORCE recommendation)	35 (89.7)	18 (81.8)	0.443‡
Other	4 (10.3)	4 (18.2)	

* Percentages reported out of the number of the participants in each sub-group (‘Yes’ or ‘No’) of those who worked on or recruited to the study.

† Chi-squared test.

‡ Fisher’s exact test.

FORCE, FOrearm Fracture Recovery in Children Evaluation.

Of those aware of the dissemination materials, the vast majority agreed or strongly agreed that they helped to raise awareness of the study findings (n = 56, 91.8%). Similar numbers agreed or strongly agreed that the dissemination materials meant the study findings were more likely to be implemented (n = 52, 85.2%) and were easy for clinicians to understand (n = 59, 96.7%).

## Discussion

This survey offers insight into the factors that influence whether findings from a large, high-quality clinical trial are adopted into routine practice. While the FORCE trial clearly demonstrated that torus fractures in children can be safely managed with a soft bandage or no immobilization and immediate discharge, this survey highlights that publication of evidence alone is insufficient to drive consistent change in clinical behaviour. Practice change appears to be shaped by a combination of clinician engagement, social influence, and practical support for implementation.

Clinicians who were involved in delivering or recruiting to the FORCE trial were more likely to report adopting both the recommended treatment and follow-up strategy. Previous research has focused on barriers to implementation, yet few studies have examined whether participation in research increases adoption of new evidence.^13^ Our finding suggests that active participation in research may increase confidence in the evidence and a sense of ownership over the resulting practice change. The findings from FORCE have clear benefits for children and their families, as well as healthcare systems, notably in reducing unnecessary appointments and clinical resource use.^14^ Involvement in research may also reduce uncertainty and resistance by allowing clinicians to observe outcomes directly and engage with questions of safety and equivalence in real time. This supports growing literature within implementation science that engagement in research can act as a powerful enabler of knowledge translation.^15^

Beyond formal trial involvement, peer-to-peer influence emerged as an important driver of change, which is reflected in the wider literature.^16^ Many respondents cited conversations with colleagues as a key factor in overcoming resistance, particularly when confronting entrenched practices, such as routine immobilization. These findings suggest that practice change is socially mediated, with informal professional networks playing an important role in legitimizing it, particularly where new evidence challenges established practice.

The availability and use of targeted dissemination resources were key facilitators of implementation. Respondents who reported using the FORCE dissemination materials were more likely to manage patients in line with trial recommendations. These resources, particularly patient- and parent-facing materials, appear to have reduced barriers at the point of care by supporting conversations with families, addressing safety concerns, and standardizing explanations. Such tools may also reduce the cognitive and organizational burden of changing practice, particularly where updating local guidelines or pathways is time-consuming.

The barriers to implementation that our study identified were largely consistent with previous implementation work^16-26^ and included resistance from colleagues, clinical support staff, and patient or carer expectations. Free-text responses provided insight into how guideline ownership and professional roles influenced the pace of change. Several respondents described slow progress in implementation when guidelines, which were intended primarily for use in the ED, were led by clinicians outside the ED. Resistance was also described among staff whose routine roles involved plaster application, suggesting that implementation can disrupt established professional roles as well as clinical routines. These findings suggest that implementation efforts should engage the entire care pathway, including non-medical staff, and that change may be more readily achieved when driven by the clinical teams most directly responsible for patient management.

Our findings reinforce a key message that the work of research does not end with publication. Evidence is most likely to change practice when trials are designed with implementation in mind, by promoting broad clinician involvement in research delivery and providing high-quality, ready-to-use dissemination materials following the study completion. For relatively simple interventions with clear patient and system benefits, such as the management of torus fractures, proactive implementation strategies may accelerate uptake, reduce unwarranted variation, and minimize research waste.

This survey-based study has several limitations. Most notably, the individual response rate was modest (13.2%), although responses represented 47 of 76 PERUKI sites (61.8%), providing broad departmental-level data. As with all voluntary surveys, there is a risk of response bias, and clinicians who were more aware of the trial or more engaged in evidence-based practice may have been more likely to respond. In addition, reported practice was self-declared and not independently verified against clinical records.

As PERUKI sites are research-active PEDs, the findings may not be generalizable to other settings. Furthermore, non-responding departments may have had limited awareness of the trial or, conversely, may have already implemented changes and therefore perceived little value in responding. As such, the true level of national implementation remains uncertain and may be higher or lower than reported within the study. Future work could strengthen the assessment of national uptake through targeted follow-up with non-responding sites, analysis of local protocols, and ultimately evaluating clinical records.

In conclusion, this survey demonstrates that the findings of the FORCE trial have changed practice across the UK, with implementation in many PEDs. Implementation appeared most successful where clinicians were involved in the original research and had access to implementation tools. These findings support the integration of implementation strategies, such as multimedia tools and template guidelines, into the design and delivery of clinical trials. Early consideration of implementation strategies may reduce research waste, accelerate evidence uptake, and promote more consistent and evidence-based care, supporting the theory that you can FORCE research implementation.

**Take home message**

- The Forearm Fracture Recovery in Children Evaluation (FORCE) study demonstrated that torus fractures of the distal radius in children can safely be managed with a soft bandage/no immobilization and immediate discharge, yet this survey found that publication of strong evidence did not itself drive adoption.

- Clinicians involved in the original FORCE study research, or who reported peer influence from colleagues, were significantly more likely to have implemented the change in practice.

- Access to practical dissemination (e.g. patient information leaflets, web materials, departmental protocols) were associated with improved implementation, suggesting these are useful tools when translating trial evidence into routine care.

## Data Availability

The data that support the findings for this study are available to other researchers from the corresponding author upon reasonable request.
